# Enhancement of Intermediate-Temperature Strength of Corundum-Spinel Castables via Incorporation of Zn(OH)_2_ Powders

**DOI:** 10.3390/ma18122777

**Published:** 2025-06-12

**Authors:** Yifan Dong, Mantang He, Mengyang Sang, Xin Qiu, Pengyu Xu, Xinhong Liu, Quanli Jia

**Affiliations:** Henan Key Laboratory of High Temperature Functional Ceramics, School of Materials Science and Engineering, Zhengzhou University, Zhengzhou 450001, China; dyifan2024@gs.zzu.edu.cn (Y.D.); hemantang111@gmail.com (M.H.); sangmengyang@gs.zzu.edu.cn (M.S.); qx15639052049@163.com (X.Q.); liuxinhong@zzu.edu.cn (X.L.)

**Keywords:** corundum-spinel castables, purging plug, Zn(OH)_2_, intermediate-temperature strength

## Abstract

Corundum-spinel based purging plugs are extensively employed in steel ladle refining processes. Traditionally, these plugs are manufactured through a high-temperature firing process that not only demanded substantial energy consumption but also resulted in a dense microstructure with higher strength; however, they often led to undesirable consequences such as fracture and thermal spalling, significantly impeding the enhancement of their service life. In recent years, the steel industry has witnessed the emergence of unfired purging plugs as an alternative solution. Unfortunately, there are some shortcomings including low strength at intermediate-temperature and poor volume stability, which easily lead to a short life and lower blowing rate of the unfired purging plug, thereby restricting their utilization. Aiming to improve the intermediate-temperature properties of the unfired purging plug, the effect of Zn(OH)_2_ on the properties of the castables was investigated. The results show that the cold strength of the specimens sintered at different temperatures remarkably increased with rising Zn(OH)_2_ content, for instance, CMOR values of the specimens sintered at 800 °C escalated from 3.19 MPa to 14.98 MPa. Furthermore, the incorporation of Zn(OH)_2_ led to a reduction in permanent linear change and a marked increase in hot strength. The remarkable improvement in intermediate-temperature strength can be attributed to the formation of ZnCr_2_O_4_ and ZnAl_2_O_4_ spinel phases originating from the reaction between ZnO derived from the decomposition of Zn(OH)_2_, and the existing Cr_2_O_3_ or Al_2_O_3_. These spinel phases create a reinforcing effect, thereby substantially enhancing the mechanical properties of the specimens after firing at intermediate temperatures.

## 1. Introduction

To date, high-performance steel products have been utilized in many areas and are increasingly on the rise; therefore, they need to be further improved by long-time refining steel metallurgical processes. As important functional parts, purging plugs played a curious role on the refining process in steel ladle [1,2,3,4]. Pre-fired corundum-spinel purging plugs have been commonly utilized in refining steel ladles because of their higher cold and high-temperature strength, good slag resistance and volume stability [5,6,7,8], furthermore, their hot strength and slag resistance can be further enhanced by adding Cr_2_O_3_ powders. However, these pre-fired purging plugs also possessed some shortcomings including higher energy consumption derived from firing at a high temperature (>1500 °C), longer production period, poorer thermal shock resistance, etc. In addition, cracks, fracture or thermal spalling easily occurred because of their dense microstructure and higher strength, which noticeably restricted their service life improvement, thereby limiting the enhancement of steel ladle heats [7,8]. To overcome these shortcomings, many studies have been conducted, such as using modified aggregates (resin coated alumina [9], alumina bubble [10], bonite [11,12], zirconia-alumina aggregates [13]), different types of spinel [14,15], etc.; however, their properties still needed to be enhanced.

Recently, calcium aluminate cement (CAC)-bonded corundum-spinel purging plugs (CSPPs) have been fabricated at a lower temperature (about 600 °C) [5,16]. However, they also have some shortcomings including low strength at intermediate-temperature, poor volume stability, which easily led to a shorter service life and lower blowing rate, thereby restricting their application in steel ladle refining processes [17,18,19]. Previous works [20,21,22] found that intermediate-temperature strength of corundum castables can be notably enhanced via incorporating Zn(OH)_2_. Furthermore, their hot strength was also increased, which was attributed to the formation of ZnAl_2_O_4_ in the matrix, creating a strengthening effect. In addition, thermal shock resistance of the specimen was also slightly enhanced because small pores were generated by the decomposition of Zn(OH)_2_. Our previous work demonstrated that CAC bonded CSPPs with ZnO addition also possessed higher physical properties and high-temperature properties [16].

Aiming to enhance the intermediate-temperature strength and volume stability of unfired CSPPs, Zn(OH)_2_ powders were applied as an additive to CSPPs. The effects of firing temperatures and Zn(OH)_2_ content on the physical properties, hot strength and thermal shock resistance, phase compositions, and microstructures of CSPPs were investigated. Results show that the cold strength of the CSPPS after heat treatment at 600–1600 °C was remarkably enhanced, and strength improvement mechanisms were attributed to forming ZnCr_2_O_4_ and ZnAl_2_O_4_ spinel phases at 600–1000 °C, and (Mg_1−x_Zn_x_)Al_2_O_4_ or Mg(Al,Cr)_2_O_4_ spinel solid solution were performed at 1400–1600 °C, creating an enforcing effect on the strength improvement.

## 2. Experimental

Tabular alumina aggregates (6–0.08 mm, from Almatis, Qingdao, China), fused corundum powders (<0.088 mm, from Kaifeng Tenai Co., Kaifeng, China), fused MgAl_2_O_4_ spinel powders (<0.044 mm, from Kaifeng Hecheng Co., Kaifeng, China), ultrafine alumina (D50 = 1.2 μm, from Kaifeng Tenai Co., China), chromia powders (Cr_2_O_3_: 99%, <20 μm, from Luoyang Zhengjie Co., Luoyang, China), calcium aluminate cement (CAC, Secar 71, from Imerys Co., Shanghai, China), and Zn(OH)_2_ fines (AR, <0.044 mm, from Aladdin Co., Shanghai, China) were utilized as starting materials, while Castment FS 10 was selected as the dispersant. The ratio of aggregates and matrices in CSPPS was 70:30, and the samples with various Zn(OH)_2_ additions (0, 0.3%, 0.6%, 0.9% and 1.2%) were labeled sample ZH0, ZH1, ZH2, ZH3, ZH4, respectively. The castables matrix ZH4 without spinel fines, labeled as sample ZH5, was also investigated as a reference sample, which were presented in Table 1.

The castable mixtures were blended with 4.20% water and cast into 25 mm × 25 mm × 150 mm bars, thereafter they were cured at room temperature and dried at 110 °C. Finally, the samples were heated at 600 °C, 800 °C, 1000 °C, 1400 °C, and 1600 °C for 3 h, respectively. Physical properties including apparent porosity (A.P.), bulk density (B.D.), cold modulus of rupture (CMOR), cold crushing strength (CCS), and permanent linear change (PLC) of the samples after firing at different temperatures were conducted according to Chinese standards. Thermal shock resistance (TSR) of the specimens pre-fired at 1000 °C and 1600 °C was measured by air-cooling (ΔT = 1100 °C, 3 cycles), and retained strengths were tested and residual CMOR ratios of the samples were calculated. The hot modulus of rupture (HMOR) of the bar specimens after drying at 110 °C was tested with a 3-point bending technique at 1400 °C for 1 h. Crystalline phases and microstructures of pre-firing samples were characterized using X-ray diffraction (XRD, Philips Xpert, Cu Kα), scanning electron microscope (SEM, Zeiss EVO H15, Jena, Germany), and energy dispersive spectrum (EDS, INCA X-act) analysis.

## 3. Results and Discussion

**Figure 1:**
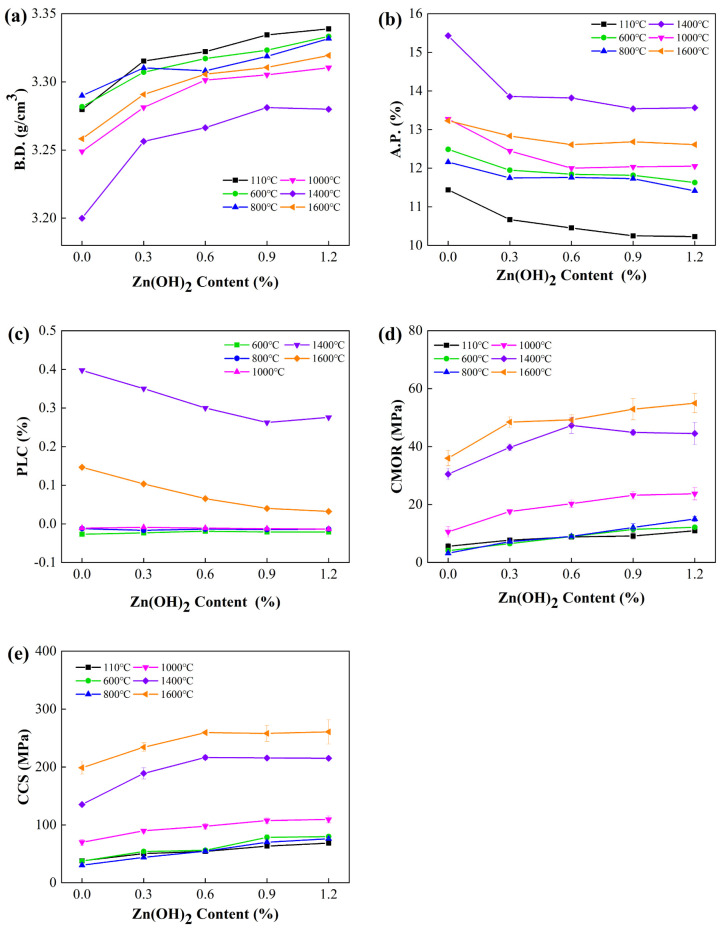
Variation in physical properties as a function of Zn(OH)_2_ content. (**a**) B.D., (**b**) A.P., (**c**) PLC, (**d**) CMOR, and (**e**) CCS.

Influences of Zn(OH)_2_ content and firing temperature on the physical properties of the samples are illustrated in Figure 1. As the Zn(OH)_2_ content increased from 0 to 1.20%, A.P. values of the samples heat treatment at 110–1600 °C gradually decreased, while their B.D. values increased. In particular, A.P. values of the samples after firing at 110 °C, 1000 °C, and 1400 °C noticeably decreased from 11.44% to 10.23%, from 13.28% to 12.05%, and from 15.43% to 13.57%, respectively. Their PLC exhibited minimal variation at firing temperatures ≤ 1000 °C, but decreased from 0.40% to 0.26% at 1400 °C. As shown in Figure 1d, the CMOR values of the samples with higher Zn(OH)_2_ content presented a substantial improvement. The CMOR value of the samples rose remarkably from: 5.56 MPa to 10.96 MPa at 110 °C, 4.05 MPa to 12.13 MPa at 600 °C, 3.19 MPa to 14.98 MPa at 800 °C, 10.56 MPa to 23.72 MPa at 1000 °C, 30.50 MPa to 41.97 MPa at 1400 °C, 35.96 MPa to 54.97 MPa at 1600 °C, respectively. Their CCS values also increased greatly (Figure 1e), which followed a comparable trend to that of CMOR values.

As shown in Figure 1, it was found that the CMOR value of sample ZH0 exhibited a sharp decline from 5.56 MPa at 110 °C to 4.05 MPa at 600 °C, and further down to 3.19 MPa at 800 °C. This reduction in cold strength may be attributed to the decomposition of CAC hydrates, coupled with the absence of the new bonding phase formation in the matrix [15,16]. However, the cold strength (CMOR and CCS values) of the samples containing Zn(OH)_2_ after firing at 600–800 °C was remarkably enhanced, which were much higher than that of at 110 °C. These findings presented that cold strength of the specimens at 600–800 °C was dramatically enhanced via Zn(OH)_2_ addition. This finding was noticeably different from that of Zn(OH)_2_-containing spinel-free corundum castables [20], which may be attributed to the newly formed ZnCr_2_O_4_ bonding phases in the castables containing Cr_2_O_3_. The CMOR value of sample ZH4 fired at 1400 °C and 1600 °C was 37.61%, 52.86% higher than that of sample ZH0. The reasons for this will be discussed in following section.

**Figure 2:**
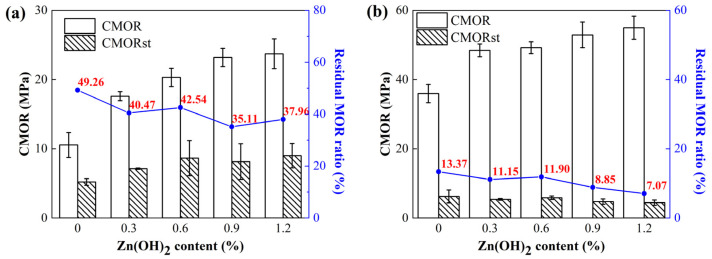
TSR of samples after treating at (**a**) 1000 °C and (**b**) 1600 °C.

The TSR of the samples after firing at 1000 °C and 1600 °C were conducted by air-cooling at 1100 °C. Residual CMOR values and residual CMOR ratios of the samples ZH0–ZH4 are revealed in Figure 2. Residual CMOR values depicted a slight decline with rising Zn(OH)_2_ content from 0 to 1.20%. Specifically, the CMOR retention ratios presented a decrease from 49.26% to 33.75% in samples pre-fired at 1000 °C and from 13.37% to 7.07% for those pre-fired at 1600 °C, indicating a marginal degradation in TSR with Zn(OH)_2_ addition. This negative effect on the TSR of the samples may be attributed to their lower A.P., denser microstructure and higher strength, thereby degrading the TSR of Zn(OH)_2_-bearing castables.

**Figure 3:**
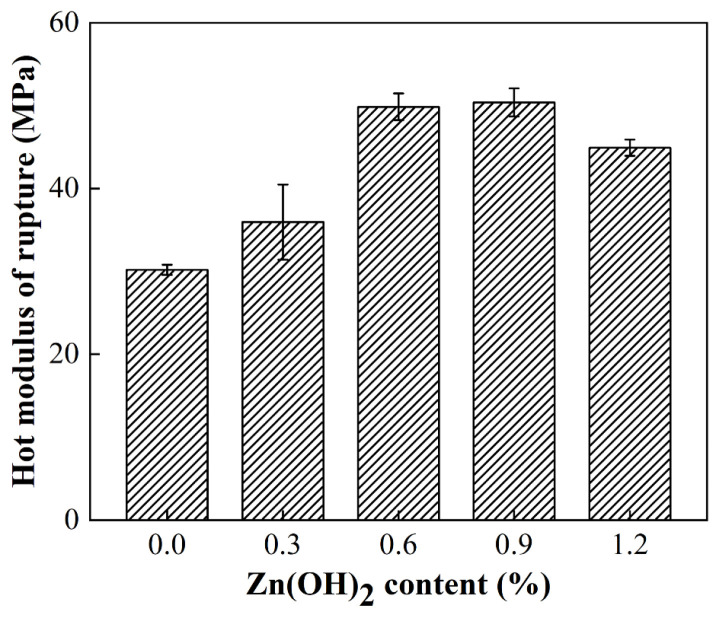
HMOR of the samples tested at 1400 °C for 1 h.

Figure 3 illuminates the HMOR values at 1400 °C of the samples ZH0–ZH4 as a function of Zn(OH)_2_ amount. On the increasing Zn(OH)_2_ contents (0→1.20%), the HMOR value of the samples noticeably enhanced, which was 30.19 MPa, 35.95 MPa, 49.85 MPa, 50.39 MPa, and 44.92 MPa, respectively, for sample ZH0, ZH1, ZH2, ZH3, and ZH4. They confirmed that the high-temperature strength of the samples was enhanced via adding Zn(OH)_2_ powders. It is noted that HMOR values at 1400 °C were higher than the cold strength of the samples pre-fired at 1400 °C, which was similar to that of nitride-bonded SiC materials [23]. The reasons can be ascribed to the fact that the hot strength is mostly controlled by crystal effect rather than glass effect [24]. After cooling, thermal expansion mismatch between corundum aggregates and spinel generates stresses and microcracks. These may weaken the structure, thereby slightly decreasing the cold strength. However, the microcracks may heal via thermal expansion of the aggregates and matrix via a thermally induced crack-healing mode. Therefore, hot strength is enhanced, which is the reason why the HMOR at 1400 °C is higher than that of the cold strength of the samples after firing at 1400 °C [24].

**Figure 4:**
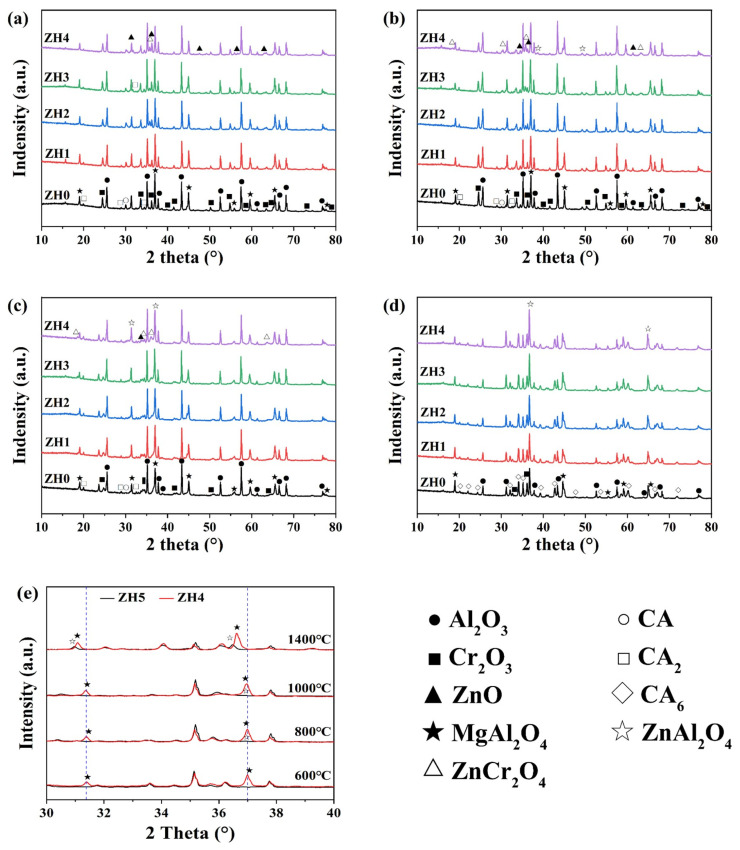
XRD patterns of the samples after treating at (**a**) 600 °C, (**b**) 800 °C, (**c**) 1000 °C, and (**d**) 1400 °C, and (**e**) comparison with the sample ZH4 and sample ZH5.

Physical properties, HMOR, and TSR of the specimens were significantly influenced by Zn(OH)_2_ addition, which can be attributed to changing in their crystalline phases and microstructures. Therefore, we further investigated them using XRD and SEM analysis. Figure 4 depicts the crystalline phases in castable matrices with varying Zn(OH)_2_ content after heat treatment from 600 to 1400 °C. At 600 °C (Figure 4a), the main phases were corundum, MgAl_2_O_4_, and Cr_2_O_3_, with minor phases of CA (CaO·Al_2_O_3_), CA_2_ (CaO·2Al_2_O_3_), and ZnO (from Zn(OH)_2_ decomposition), and trace ZnAl_2_O_4_ was also detected in Zn(OH)_2_-containing samples. The phase compositions were similar to that of at 800 °C, ZnAl_2_O_4_ peaks were detected in sample ZH4 (Figure 4b). By 1000 °C (Figure 4c), ZnO peaks disappeared completely, while ZnAl_2_O_4_ peaks intensified significantly. At 1400 °C (Figure 4d), ZnCr_2_O_4_ disappeared, and CA and CA_2_ phases vanished; they were transformed to CA_6_. Compared to sample ZH4, ZnAl_2_O_4_ peaks can be detected in sample ZH5 after firing at 600–1000 °C, and no MgAl_2_O_4_ peaks were found. Furthermore, the displacement of MgAl_2_O_4_ peaks in sample ZH4 after firing at 1400 °C shifted from 37.09° to 36.66°, indicating that (Mg_1−x_Zn_x_)Al_2_O_4_ or Mg(Al,Cr)_2_O_4_ spinel solid solution were generated at 1400 °C. This can be attributed to ZnAl_2_O_4_ or ZnCr_2_O_4_ dissolving into the MgAl_2_O_4_ spinel structure to form spinel solid solution at elevated temperatures [16,20,21,22]. These solid solutions are beneficial for enhancing the cold and hot strength of the Zn(OH)_2_-bearing castables.

**Figure 5:**
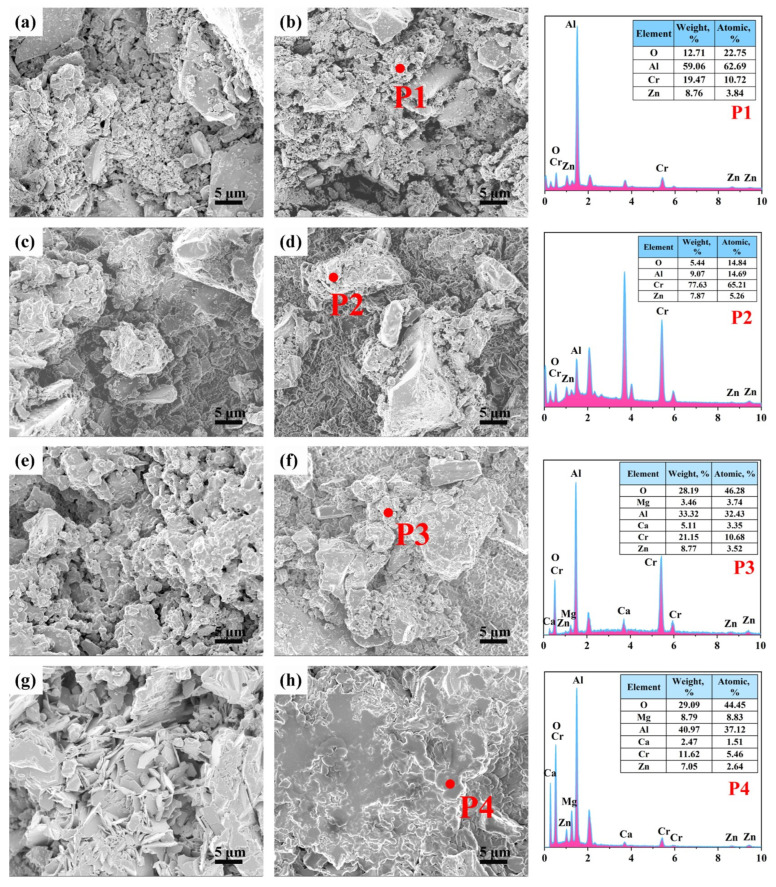
SEM images of matrix samples ZH0(**a**,**c**,**e**,**g**) and ZH2(**b**,**d**,**f**,**h**) after treating at different temperatures: (**a**,**b**) 600 °C, (**c**,**d**) 800 °C, (**e**,**f**) 1000 °C, (**g**,**h**) 1400 °C. And EDS spectrum of points 1–4.

Figure 5 demonstrates the microstructural evolution and corresponding EDS analysis of sample ZH2, sintered at 600–1400 °C. At 600 °C (Figure 5a,b), granular aggregates were observed in the ZH0 matrix, and EDS analysis of point 1 (Figure 5(P1)) revealed that the presence of Al, O, Cr, and Zn was detected. This may be owing to ZnO incorporated into the Cr_2_O_3_ and Al_2_O_3_ grains. Combined with XRD results, these aggregates likely comprised Al_2_O_3_, ZnCr_2_O_4_, and residual ZnO, explaining the reason why the cold strength of the Zn(OH)_2_-containing samples was higher than sample ZH0. Similar aggregated particles were observed at 800 °C (Figure 5c,d). EDS analysis of point 2 (Figure 5(P2)) confirmed that these particles contained ZnAl_2_O_4_ and ZnCr_2_O_4_, consistent with the XRD analysis. This demonstrates that the in situ formation of ZnAl_2_O_4_ and ZnCr_2_O_4_ contributed to improving their physical properties. Increased density and visible sintering necks can be found in the samples after tfiring at 1000 °C (Figure 5e,f), indicating particles bonding content was enhanced. These particles comprised Al, O, Mg, Ca, Cr, and Zn (Figure 5(P3)). At 1400 °C (Figure 5g), the plate-like CA_6_ grains formed an interconnected network. The particles were well sintered, and this microstructure provided a substantial strengthening, resulting in marked improvements in both cold and hot strength of sample ZH2 after treating at 1400 °C. EDS analysis depicted that they were composed of Al, O, Mg, Ca, Cr, and Zn (Figure 5(P4)), an indication that (Mg,Zn)Al_2_O_4_ or Mg(Al,Cr)_2_O_4_ spinel solid solutions was found, which matched well with the XRD analysis. The bonding content between corundum and MgAl_2_O_4_ spinel in the matrix was intensified via the formation of spinel solid solutions, which contributed to augmenting their cold and hot strength [16,20,25].

As the firing temperature increased, ZnCr_2_O_4_ and ZnAl_2_O_4_ formed from reactions between ZnO (decomposition of Zn(OH)_2_) and Al_2_O_3_ and Cr_2_O_3_ dispersed in the matrix fired at 600 °C to 1000 °C. These newly formed ZnCr_2_O_4_ and ZnAl_2_O_4_ can link among the corundum and spinel particles in the matrix, which can compensate for the strength loss as a result of decomposition of CAC hydration products. Therefore, the bonding degree of the matrix was strengthened, thereby improving cold strength of the sample at 600–1000 °C. At 1400–1600 °C, flake CA_6_ grains were formed by the reaction of Al_2_O_3_ and CA_2_, while ZnAl_2_O_4_ and ZnCr_2_O_4_ particles interspersed within the plate-like CA_6_ and spinel structure to generate an interwoven structure. The interface bonding strength between particles was greatly improved, thereby notably enhancing the strength of the castables containing Zn(OH)_2_) [16,20,25,26].

## 4. Conclusions

The corundum-spinel castables were prepared using tabular alumina aggregates, corundum fines, fused spinel fines, ultrafine alumina, and calcium aluminate cement as the matrix. To enhance the intermediate-temperature strength of the castables, Zn(OH)_2_ fines were incorporated as an additive, and the effects of Zn(OH)_2_ content on the phase composition, microstructure, and properties of the castables were systematically investigated. The results are as follows:

(1) The bulk density and cold strength of the specimens increased significantly with rising of Zn(OH)_2_ content and increasing of heat-treatment temperature. Apparent porosity decreased, and PLC gradually decreased after firing at 1400–1600 °C. HMOR values also presented a notable improvement. These may be ascribed to enhanced sintering densification behavior induced by incorporating Zn(OH)_2_, which decreased porosity and increased bulk density; therefore, their physical properties were enhanced.
(2) The enhanced intermediate-temperature strength of Zn(OH)_2_-containing castables after firing at 600–1000 °C was attributed to in situ formation of ZnAl_2_O_4_ and ZnCr_2_O_4_ bonding phases in the matrixes. These newly formed ZnAl_2_O_4_ and ZnCr_2_O_4_ mixed phases created a reinforcing effect that outweighs the strength reduction from CAC hydrate decomposition. In addition, the strengthening effect intensified with rising heat-treatment temperature, which could be the reason for the strength improvement.

## Figures and Tables

**Table 1:** Formulation of the castables matrix with Zn(OH)_2_ powders addition (wt.%).

Raw Materials	Mass Ratio (%)					
	ZH0	ZH1	ZH2	ZH3	ZH4	ZH5
Corundum powders	5	4.70	4.40	4.10	3.80	13.80
MgAl_2_O_4_ powders	10	10	10	10	10	0
α-Al_2_O_3_ ultrafine	8	8	8	8	8	8
Cr_2_O_3_ powders	3	3	3	3	3	3
CAC	4	4	4	4	4	4
Zn(OH)_2_ powders	0	0.30	0.60	0.90	1.20	1.20

## Data Availability

The original contributions presented in this study are included in the article. Further inquiries can be directed to the corresponding authors.
